# Adhesive Hydrogel Loaded with Sulfonated Chitosan Promotes Oral Mucosal Defect Repair in Diabetic Rats

**DOI:** 10.3390/bioengineering13070792

**Published:** 2026-07-10

**Authors:** Xiaohui Zhang, Gaopeng Wang, Shuwen Ding, Chenyang Luo, Jing Wang

**Affiliations:** 1 The State Key Laboratory of Bioreactor Engineering, East China University of Science and Technology, Shanghai 200237, China; 13162300673@163.com (X.Z.); 15685869612@163.com (C.L.); 2 School of Materials Science and Engineering, Engineering Research Center for Biomedical Materials of the Ministry of Education, East China University of Science and Technology, Shanghai 200237, China; chrisw834801151@outlook.com; 3 Shanghai Engineering Research Center of Tooth Restoration and Regeneration, Department of Prosthodontics Stomatological Hospital and Dental School of Tongji University, Shanghai 200072, China; elflanderald@163.com

**Keywords:** diabetic oral mucosa, photocurable hydrogel, sulfonated chitosan, wound healing, tissue engineering

## Abstract

Diabetic oral mucosal wounds exhibit impaired healing and require biomaterials with strong wet adhesion, favorable biocompatibility, and adequate mechanical stability. In this study, an in situ photocurable adhesive hydrogel (ATDS) based on sulfonated chitosan was developed for diabetic oral mucosal wound repair. ATDS exhibited a tensile strength of 50 kPa, an elongation at break of 320%, and an adhesive strength of 0.605 MPa, while also displaying a porous microstructure without obvious cytotoxicity. Compared with hyaluronic acid (HA) gel, which was completely lost by day 3, ATDS provided more durable wound coverage in the oral environment. In a diabetic rat model of oral mucosal defect, ATDS significantly accelerated wound closure, with wounds nearly completely healed by day 6, promoted re-epithelialization as early as day 3, and increased epidermis thickness by approximately 50% compared with the control group. In addition, ATDS enhanced angiogenesis and reduced the expression of the inflammatory cytokines TNF-α and IL-1β. Collectively, these findings demonstrate that ATDS effectively promotes diabetic oral mucosal wound healing through its barrier-protective, pro-angiogenic, and anti-inflammatory effects, highlighting its potential as a promising biomaterial for oral tissue engineering and regenerative applications.

## 1. Introduction

Skin and mucosa constitute the primary biological barriers of the human body and play essential roles in protecting internal homeostasis from external insults [1,2]. With the rapid increase in the aging population, the incidence of diabetes mellitus has risen substantially. The hyperglycemic microenvironment markedly impairs the repair capacity of skin and mucosal tissues, resulting in delayed wound healing and reduced quality of life [3,4,5]. In the oral cavity, diabetes further disrupts mucosal repair by altering cellular activities such as proliferation and migration [6,7].

Compared with cutaneous wounds, oral mucosal wounds are exposed to a more complex environment characterized by persistent moisture, frequent mechanical stimulation, and abundant microbial colonization, all of which hinder wound closure and re-epithelialization [8,9]. Conventional dressings often exhibit poor adhesion and limited functionality in the oral environment, highlighting the need for multifunctional wound dressings with robust wet-adhesion properties [10,11]. Adhesive hydrogels can closely conform to wound surfaces, resist salivary erosion, provide a protective barrier against contamination, and support cell adhesion and migration, thereby promoting tissue regeneration [12,13]. Consequently, the development of hydrogel dressings with strong wet adhesion, suitable mechanical properties, and multifunctional bioactivity has become an important strategy for diabetic oral mucosal repair [14,15].

Various adhesive hydrogels have been developed for wound management, including aldehyde-based adhesives, dopamine-derived systems, and N-acryloyl tris(hydroxymethyl)aminomethane (THMA)-based hydrogels [16]. Aldehyde-based adhesives achieve tissue adhesion through dynamic Schiff-base reactions. Representative oxidized dextran-based hydrogels have demonstrated tissue adhesive strengths of approximately 16 kPa on porcine skin [17]. However, their adhesive performance is highly dependent on reaction conditions, while the dynamic imine bonds formed during crosslinking may exhibit limited stability under wet physiological environments [18]. Dopamine-derived hydrogels exhibit substantially stronger wet adhesion, reaching tissue adhesive strengths of up to 153 kPa on porcine skin, but their long-term adhesive performance is often compromised by catechol oxidation [19]. THMA-based hydrogels rely primarily on abundant hydroxyl groups to establish hydrogen-bond-mediated adhesion, typically achieving adhesive strengths of approximately 7.5 kPa [16]. Nevertheless, their relatively weak mechanical properties limit their application in highly dynamic tissues, such as the oral mucosa, and additional reinforcement through composite or double-network designs is often required [20]. To overcome these limitations, polysaccharide-based hydrogels have attracted increasing attention as versatile platforms for constructing multifunctional adhesive biomaterials. Owing to their excellent biocompatibility, tunable chemical structures, and abundant functional groups—including hydroxyl, amino, and carboxyl groups—polysaccharides can be readily functionalized to integrate multiple adhesive mechanisms while simultaneously improving mechanical strength and wet tissue adhesion [21,22].

Delayed diabetic wound healing is mainly associated with persistent inflammation, impaired angiogenesis, and dysfunction of repair-related cells [23,24]. Prolonged inflammatory responses in diabetic wounds lead to sustained expression of pro-inflammatory cytokines such as TNF-α and IL-6, thereby establishing a chronic inflammatory microenvironment [25,26]. Meanwhile, hyperglycemia-induced microvascular dysfunction suppresses angiogenesis and tissue perfusion, further impairing tissue regeneration [26]. In addition, high-glucose conditions adversely affect fibroblasts and keratinocytes, resulting in defective re-epithelialization and delayed healing [27,28]. Therefore, hydrogel dressings capable of simultaneously regulating multiple pathological processes are highly desirable [29]. Recent advances have demonstrated that multifunctional hydrogels integrating anti-inflammatory, antioxidant, antibacterial, angiogenic, and glucose-regulatory properties can significantly enhance diabetic wound repair [30,31,32,33].

Sulfonated chitosan (SCS), a heparin-mimetic polysaccharide, has recently attracted considerable attention because of its ability to regulate angiogenesis and inflammatory responses by mimicking heparan sulfate proteoglycans [34,35,36]. Based on these properties, we developed a photocurable multifunctional hydrogel composed of methacrylated alginate (AlgMA), N-acryloyl tris(hydroxymethyl)aminomethane (THMA), 3-methacryloyl dopamine (DMA), and methacryloylated sulfonated chitosan (SCS-MA), designated as ATDS hydrogel. In this system, AlgMA provides structural support, THMA and DMA enhance wet adhesion, and SCS-MA contributes both bioactivity and photocrosslinkability. The resulting hydrogel exhibited favorable wet adhesion, suitable mechanical performance, and low cytotoxicity. Furthermore, in a diabetic rat oral mucosal defect model, ATDS hydrogel significantly accelerated wound healing and tissue regeneration.

## 2. Materials and Methods

### 2.1. Raw Materials and Reagents

Chitosan with a deacetylation degree greater than 95% (Mw ≈ 300,000) was purchased from Shenzhen Zhongfayuan Biotechnology Co., Ltd. (Shenzhen, China). Methacrylated alginate (AlgMA, EFL-AlgMA-50K, Mw ≈ 50,000 Da, with a methacrylation grafting ratio of 20–40%) was purchased from Suzhou Yongqinquan Intelligent Equipment Co., Ltd. (Suzhou, China). Lithium phenyl-2,4,6-trimethylbenzoylphosphinate (LAP, AR) was purchased from Huaxia Siyin Shanghai Biotechnology Co., Ltd. (Shanghai, China). Acrylamide (AR) was purchased from Shanghai Aladdin Biochemical Technology Co., Ltd. (Shanghai, China). N-Acryloyl tris(hydroxymethyl)aminomethane (THMA), 3-methacryloyl dopamine (DMA), and methacrylic anhydride were purchased from Ron Reagents (Shanghai, China). Hyaluronic acid gel (HA, Mw ≈ 1.3 × 10^5^ Da) was obtained from Shanghai Qisheng Biological Preparation Co., Ltd. (Shanghai, China). The TNF-α ELISA Kit and IL-1β ELISA Kit were purchased from NeoBioscience (Shenzhen Xinbosheng Biotechnology Co., Ltd., Shenzhen, China). CD31 and α-SMA antibodies were purchased from Wuhan Servicebio Technology Co., Ltd. (Wuhan, China). The Fourier-transform infrared spectroscopy device (FTIR, Nicolet iS50) was purchased from Thermo Fisher Scientific (Waltham, MA, USA). The superconducting Fourier-transform nuclear magnetic resonance spectrometer (AVANCE III 400) was purchased from Bruker Corporation (Billerica, MA, USA).

### 2.2. Preparation of Methacryloylated Sulfonated Chitosan

Sulfonated chitosan was synthesized as previously reported [37,38]. Briefly, 0.5 g of sulfonated chitosan was dissolved in 10 mL of ultrapure water to obtain a 5 wt% aqueous solution. To this solution, N,N-dimethylformamide (DMF) was added dropwise to a 5 wt% sulfonated chitosan aqueous solution at a water/DMF volume ratio of 3:2 under stirring. After cooling to 4 °C, 500 μL of methacrylic anhydride was slowly added. The pH of the reaction mixture was adjusted to 8.0–8.2 using 10 M NaOH. During the first 4 h of the reaction, the pH was measured every 30 min, and if any deviation from the range of 8.0–8.2 was observed, pre-cooled 10 M NaOH was added dropwise to readjust the pH. After this 4 h active maintenance period, the mixture was incubated at 4 °C overnight without further pH adjustment. The resulting solution was precipitated in excess anhydrous ethanol, and the precipitate was redissolved in ultrapure water and dialyzed (MWCO 14 kDa) against ultrapure water for 3 days at pH 7–8. The purified product was lyophilized at −80 °C for 2 days to obtain methacryloylated sulfonated chitosan (SCS-MA). Successful methacrylation was confirmed by Fourier-transform infrared spectroscopy (FTIR) and proton nuclear magnetic resonance (^1^H NMR).

### 2.3. Preparation of Hydrogels

Methacrylated alginate (AlgMA) was dissolved in water at 0.75 wt%. N-acryloyl tris(hydroxymethyl)aminomethane (THMA), methacryloylated sulfonated chitosan (SCS-MA), 3-methacryloyl dopamine (DMA), and LAP (0.5 wt%) were subsequently added to the AlgMA solution. The precursor solutions were injected in situ and photocrosslinked under UV irradiation (300–500 nm) for 10 min to form hydrogels [39,40]. The compositions of THMA, AlgMA, DMA, SCS-MA, and LAP are summarized in Table 1. Among the prepared formulations, ATDS containing 25 wt% SCS-MA (ATDS-25 wt%) was selected for subsequent experiments.

### 2.4. Characterization of Mechanical Properties and Adhesion of Hydrogels

The mechanical and adhesive properties of the hydrogels were evaluated using a universal mechanical testing machine. For tensile testing, cured hydrogel strips (4 cm in length) were fixed at both ends with a clamping length of 1 cm. According to the recommendations of ASTM D882, the tests were performed at a trigger force of 0.05 N to eliminate initial slack and at a tensile speed of 300 mm/min (within the standard’s permissible range of 5–500 mm/min). Stress–strain curves were generated from the obtained data.

To evaluate the skin adhesion of the hydrogels, lap shear tests were conducted using fresh pig skin purchased from a local market. The pig skin was washed with water, and residual fat was carefully removed using a scalpel blade, followed by further cleaning with detergent to eliminate remaining grease. After blotting dry with tissue paper, the skin was dried at 60 °C for 5 min before use. Unused pig skin was stored at −20 °C for no longer than one week.

The prepared pig skin was cut into strips (4 cm × 1 cm), and a 1 cm × 1 cm adhesive area was marked on each strip. Hydrogel precursor solution was applied to the marked area using a 1000 μL pipette tip with a thickness of approximately 0.5 mm. Two pig skin strips were overlapped at the adhesive area, gently pressed together, and placed into a UV curing chamber (JD818, Guangzhou, China) equipped with four 9 W lamps (wavelength range: 300–500 nm), where the multi-lamp configuration ensured uniform irradiation from all directions across the entire adhesive joint, and the samples were cured for 10 min at the center of the chamber tray. Adhesion tests were subsequently performed at a trigger force of 0.05 N and a tensile speed of 300 mm/min. Force–displacement curves were obtained, and the maximum adhesive stress (MPa) was calculated by dividing the maximum force (N) by the adhesive area (m^2^).

### 2.5. Microstructure and Ex Vivo Adhesion of ATDS Hydrogels

The prepared ATDS hydrogels were lyophilized at −80 °C for 48 h, and their microstructure was observed using scanning electron microscopy (SEM, S-3400, Hitachi High-Technologies Corporation, Tokyo, Japan).

To further evaluate the conformal adhesion of the hydrogels to tissue surfaces, ATDS hydrogels were applied to rat dorsal skin and oral palatal tissues. Briefly, SD rats (200–250 g) were fasted for 6 h and anesthetized by intraperitoneal injection of sodium pentobarbital (100 mg/kg). After confirmation of adequate anesthesia, dorsal skin (~1 cm^2^) and oral palatal mucosal (~0.5 cm^2^) tissues were harvested, rinsed with ice-cold saline, and gently dried.

ATDS-25 wt% precursor solution was evenly applied to the tissue surface at a thickness of approximately 0.5 mm and photocrosslinked under 395 nm UV irradiation for 5–10 s. The samples were then snap-frozen in liquid nitrogen and lyophilized at −80 °C for 48 h. After freeze-drying, the samples were fractured under liquid nitrogen, and the cross-sectional morphology was examined by SEM. In addition, cryosections of tissues with adhered hydrogels were prepared and analyzed by SEM to evaluate the conformal adhesion between the hydrogel and tissue surface.

### 2.6. Measurement of Gelation Time

The gelation behavior of ATDS hydrogels was evaluated using a rotational rheometer (Thermo HAAKE MARS 60, Thermo Fisher Scientific, Waltham, MA, USA) in time sweep mode. Measurements were performed using a 25 mm parallel-plate geometry with a sample thickness of 0.2 mm. UV irradiation was applied at an intensity of 55 mW/cm^2^ (320–500 nm). During testing, UV exposure was initiated 30 s after the start of measurement and maintained for 30 s, with a total recording time of 180 s. Before gelation, the loss modulus (G″) exceeded the storage modulus (G′), whereas after gelation, G′ became greater than G″, indicating the formation of a stable hydrogel network. The intersection point of G′ and G″ was defined as the gelation time.

### 2.7. Determination of Swelling Properties

To characterize the swelling behavior, the prepared ATDS hydrogels were immersed in phosphate-buffered saline (PBS, pH = 7.4) at 37 °C for 72 h. At each predetermined time interval, the hydrogels were collected and weighed (W_1_). The initial weight of the samples (W_0_) was also recorded. The swelling ratio was calculated using the following formula:

Swelling ratio (%) = (W_1_/W_0_) × 100%

### 2.8. Cytotoxicity of ATDS Hydrogels

ATDS hydrogels were sterilized under UV irradiation for 24 h and incubated in complete DMEM high-glucose medium containing 10% fetal bovine serum and 1% penicillin–streptomycin at a ratio of 0.1 g/mL to prepare hydrogel extracts. A hydrogel composed of methacrylated alginate (AlgMA), acrylamide (AM), 3-methacryloyl dopamine (DMA), and methacryloylated sulfonated chitosan (SCS-MA) containing 25% AM monomer (AADS hydrogel) was used as a positive control, while complete medium alone served as the negative control. Extracts were collected after incubation at 37 °C with 5% CO_2_ for 24 or 72 h.

HaCaT human keratinocytes obtained from the Cell Bank of the Chinese Academy of Sciences were seeded into 96-well plates at 1 × 10^3^ cells/well. After overnight attachment, cells were cultured with hydrogel extracts or complete medium for 1 or 3 days. Cell viability was evaluated using an MTT assay (5 mg/mL in PBS, Sigma-Aldrich, St. Louis, MO, USA).

### 2.9. Induction of Diabetes Model

Diabetes was induced by a single intraperitoneal injection of streptozotocin (STZ, Aladdin). Before injection, rats were fasted for 16 h with free access to water. STZ was freshly dissolved in citrate buffer (0.1 mol/L, pH 4.2–4.4) to obtain a 2.5% solution and administered intraperitoneally at 50 mg/kg [41]. Rats were anesthetized with sodium pentobarbital (50 mg/kg, intraperitoneal injection), and adequate anesthesia was confirmed by the absence of a toe pinch reflex. Fasting blood glucose levels were measured on days 1, 3, 7, and 14 after STZ injection. Rats with fasting blood glucose > 13.3 mmol/L or random blood glucose > 16.7 mmol/L were considered diabetic. To ensure stable hyperglycemia and minimize inter-individual variation, only rats with random blood glucose levels consistently ≥ 20.0 mmol/L were included in subsequent experiments [42].

### 2.10. Establishment of Oral Mucosa Defect Model in Diabetic Rats

After successful induction of diabetes, an oral mucosal defect model was established. Rats were anesthetized by intraperitoneal injection of sodium pentobarbital (50 mg/kg) and fixed in the supine position. The oral cavity was opened using hemostatic forceps, and the palatal mucosa was disinfected with 70% ethanol and iodophor.

Circular full-thickness mucosal defects (2.5 mm in diameter) were created bilaterally in the anterior palatal rugae region posterior to the upper incisors using a dental handpiece with a round bur. The wounds involved both the epithelial layer and lamina propria.

Diabetic rats were randomly assigned to three groups (*n* = 12/group): control, hyaluronic acid gel (HA), and ATDS hydrogel groups. The control group received no treatment. In the HA group, 100 μL of HA gel was applied to each wound. In the ATDS group, 100 μL of ATDS precursor solution was injected into each wound and photocrosslinked for 1 min using a handheld curing light. HA was selected as the control because it is a major ECM component widely used as a hydrogel scaffold in wound dressings, with well-documented applications in wound healing [43,44,45]. This comparison helps evaluate the therapeutic performance and potential advantages of our proposed material.

### 2.11. Collection of Animal Samples

At predetermined time points (days 1, 3, 6, and 8), three rats from each group were randomly selected, and wound images were captured using a digital camera (Nikon, Tokyo, Japan). Rats were then euthanized, and wound mucosal tissues were harvested for subsequent analyses.

Half of the collected tissues were fixed in 4% paraformaldehyde, embedded in paraffin, and sectioned at 7 μm for hematoxylin and eosin (H&E) staining and immunofluorescence analysis. The remaining tissues were homogenized in ice-cold lysis buffer containing protease inhibitors and centrifuged at 4 °C (10,000× *g*, 10 min). The supernatants were collected for enzyme-linked immunosorbent assay (ELISA). TNF-α and IL-1β levels were quantified using ELISA kits from NeoBioscience and normalized to total protein content. Results are expressed as pg/mg total protein [46].

### 2.12. Statistical Analysis

All quantitative data are presented as mean ± SD. Statistical analyses were performed using GraphPad Prism 8.0 (USA) by two-way analysis of variance (two-way ANOVA), followed by Tukey’s multiple-comparison test. A *p*-value < 0.05 was considered statistically significant.

## 3. Results

### 3.1. Synthesis and Characterization of SCS-MA

**Figure 1:**
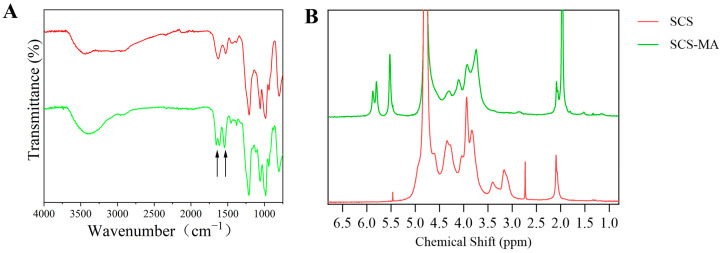
Characterization of SCS-MA: (**A**) FTIR spectra. The black arrows indicate the characteristic peaks of methacryloyl groups at 1535 cm^−1^ and 1654 cm^−1^. (**B**) ^1^H NMR spectrum.

SCS-MA exhibited characteristic methacryloyl peaks at 1535 cm^−1^ and 1654 cm^−1^ that were absent in SCS, confirming successful methacrylation of sulfonated chitosan (Figure 1A). Consistently, the ^1^H NMR spectrum showed characteristic vinyl proton peaks at 5.52 and 5.80 ppm, along with a methyl proton peak at 1.97 ppm corresponding to the methacryloyl group (Figure 1B). Collectively, these results confirmed the successful grafting of methacryloyl groups onto sulfonated chitosan chains.

### 3.2. Adhesion and Mechanical Properties of ATDS Hydrogels

**Figure 2:**
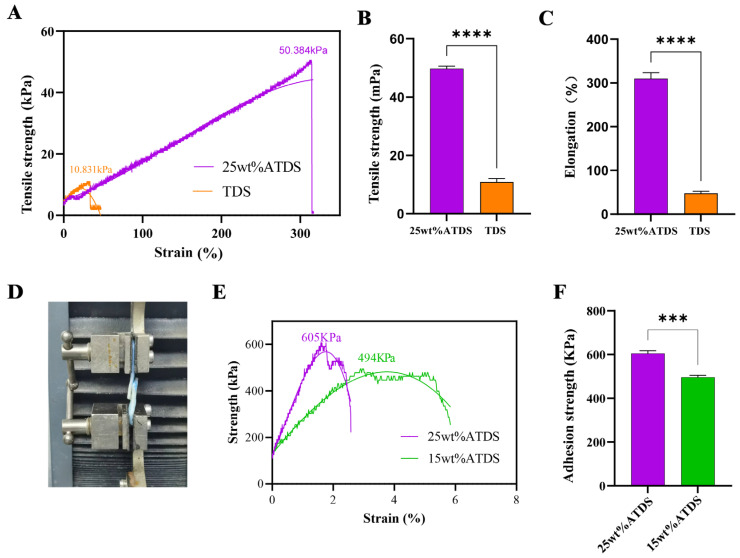
Mechanical and adhesion properties of ATDS hydrogels: (**A**) Tensile strength–strain curves of ATDS (25%) and TDS (without AlgMA) hydrogels. (**B**) Quantitative analysis of tensile strength. (**C**) Quantitative analysis of elongation at break. (**D**) Schematic of a single-lap tensile shear test setup. (**E**) Adhesive strength–strain curves of hydrogels with different THMA contents (15% vs. 25%). (**F**) Quantitative analysis of adhesive strength., *** *p* < 0.001, **** *p* < 0.0001.

The mechanical properties of the hydrogels were assessed by tensile testing, and the corresponding stress–strain curves are shown in Figure 2A. To clarify the contribution of AlgMA to the composite system, a TDS hydrogel without AlgMA was used as a control. The ATDS (25%) hydrogel exhibited a maximum tensile strength of 50 kPa and an elongation at break of 320%, whereas the TDS hydrogel showed only 10.8 kPa tensile strength and less than 40% elongation at break (Figure 2B,C). These results indicate that AlgMA markedly improves the mechanical strength and flexibility of the hydrogel, thereby enhancing its ability to withstand mechanical disturbances in the oral environment.

The adhesive properties of the hydrogels were evaluated by lap shear testing (Figure 2D). As shown in Figure 2E, hydrogel adhesion positively correlated with THMA content. Increasing the THMA content from 15% to 25% enhanced the shear adhesive strength from 0.494 MPa to 0.605 MPa, representing a 22% increase (Figure 2F). Owing to the limited solubility of THMA, the 25% formulation was selected as the optimal composition for subsequent studies.

Overall, the ATDS hydrogel exhibited favorable mechanical performance and strong tissue adhesion, highlighting its potential as an adhesive dressing for oral mucosal wound repair.

### 3.3. Microstructure and Tissue Conformability of ATDS Hydrogels

**Figure 3:**
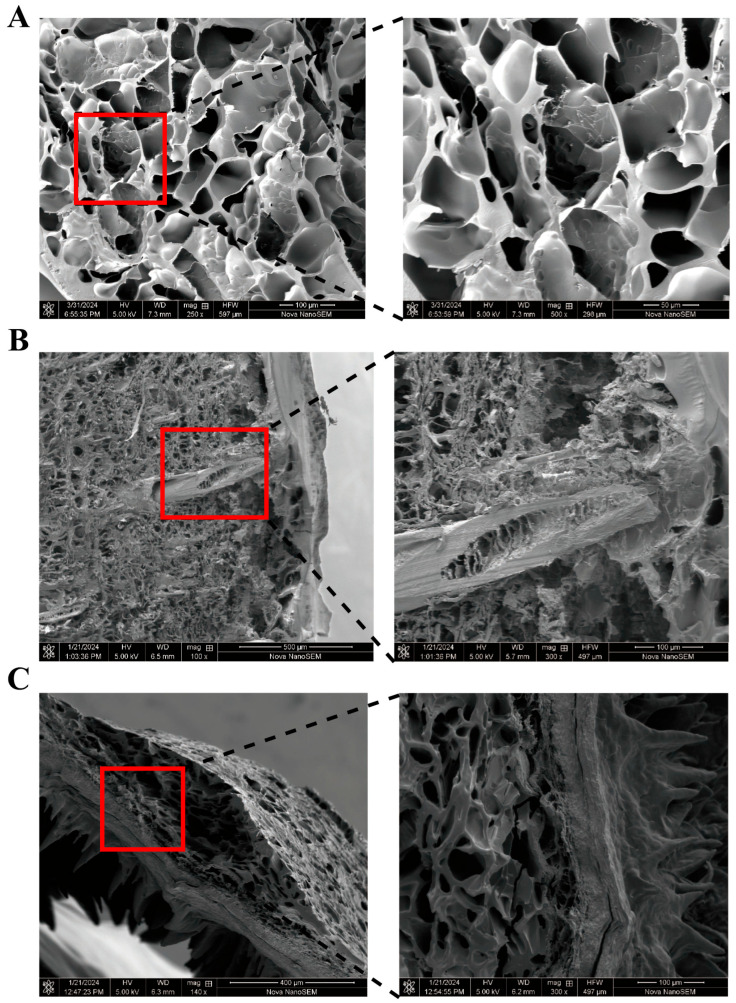
Microscopic morphology of ATDS hydrogels: (**A**) Scanning electron microscopy (SEM) image of lyophilized ATDS hydrogel. (**B**) Conformability of ATDS hydrogel to rat dorsal skin. (**C**) Conformability of ATDS hydrogel to rat palatal mucosa. Right: Enlarged view of the red-boxed region in the left panel.

Scanning electron microscopy (SEM) images at 250× magnification revealed that the lyophilized ATDS hydrogels possessed a porous microstructure, which is favorable for wound dressing applications (Figure 3A). At 500× magnification, the pore size was estimated to be approximately 50 μm based on the scale bar.

The conformability of the hydrogels to rat palatal mucosa and dorsal skin tissues was further evaluated ex vivo using cryosections. SEM images demonstrated that the ATDS hydrogel adhered closely to both dorsal skin (Figure 3B) and palatal mucosal tissues (Figure 3C). At 140× magnification, the porous hydrogel structure was observed to be firmly integrated with the tissue surface. Even at 500× magnification, minimal interfacial gaps were detected between the hydrogel and tissue, indicating excellent conformal adhesion.

These findings demonstrate that the ATDS hydrogel possesses both a favorable porous architecture and excellent tissue conformability. Such properties may provide a suitable microenvironment for cell attachment, migration, and tissue ingrowth while protecting oral wounds from fluid infiltration and mechanical irritation.

### 3.4. Swelling Properties of ATDS Hydrogels

**Figure 4:**
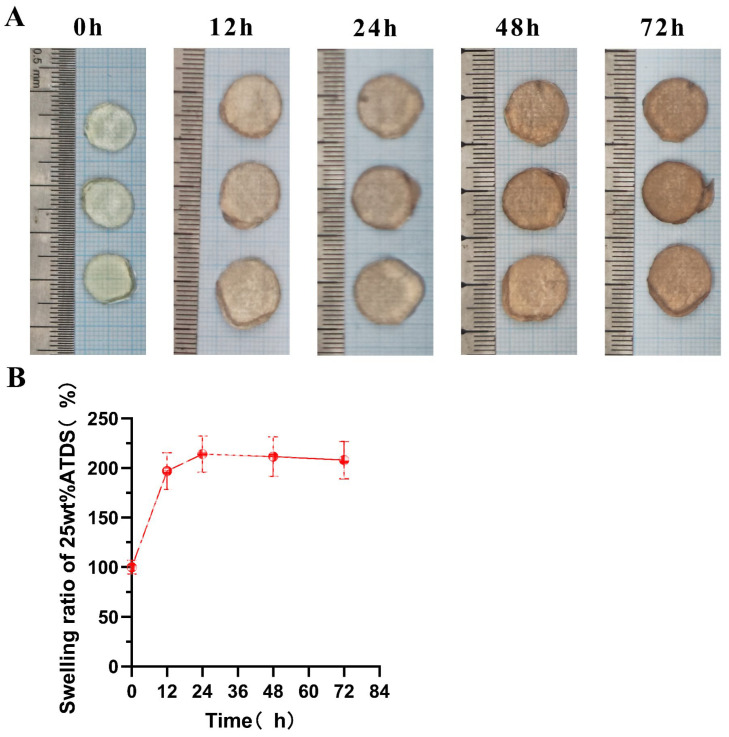
Swelling properties of ATDS hydrogels: (**A**) Photographs of hydrogel swelling at various time intervals. (**B**) Time-dependent changes in the swelling ratio of hydrogels.

The swelling behavior of ATDS hydrogels was evaluated by immersion in PBS solution (10.0 mM, pH 7.4) for 12, 24, 48, and 72 h. As shown in Figure 4A, the hydrogels gradually absorbed water and expanded in volume over time. Quantitative analysis demonstrated that the hydrogels reached swelling equilibrium within 24 h, with an equilibrium swelling ratio of approximately 214% (Figure 4B).

The moderate swelling behavior enabled the hydrogel to effectively conform to surrounding tissues while maintaining structural stability, which is beneficial for tissue integration and wound healing.

### 3.5. Gelation Time of ATDS Hydrogels

**Figure 5:**
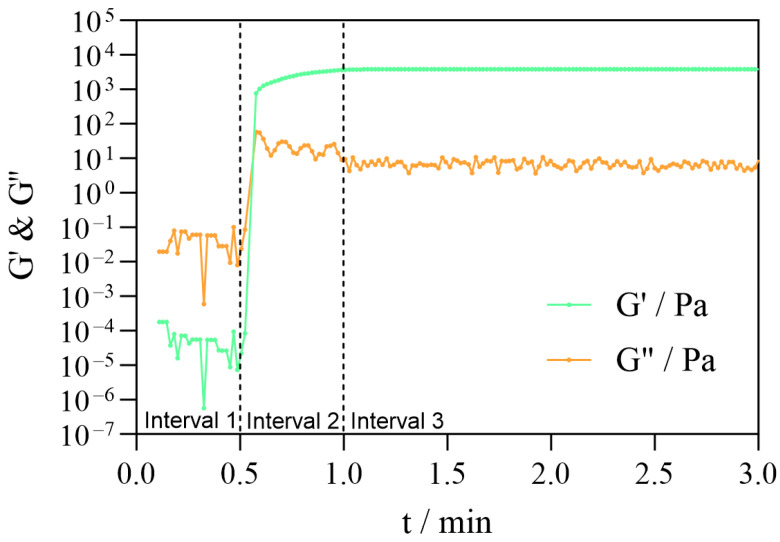
ATDS hydrogel gelation time. During testing, UV exposure was initiated 30 s after the start of measurement (at the onset of Interval 2), maintained for 30 s, and then turned off, with a total recording time of 180 s; the vertical dashed lines demarcate the three intervals.

The gelation behavior of the ATDS hydrogels was evaluated by time sweep rheological analysis using a rotational rheometer equipped with an in situ UV curing system (Mars60, Thermo Fisher Scientific, Waltham, MA, USA). As shown in Figure 5, the storage modulus (G′) rapidly exceeded the loss modulus (G″) upon UV irradiation, indicating the formation of a crosslinked hydrogel network. The precursor solution gelled within 0.57 s under UV light (300–500 nm), and the storage modulus subsequently stabilized at approximately 3800 Pa.

These results demonstrate that the ATDS hydrogel undergoes rapid photocrosslinking with an ultrashort gelation time, which is advantageous for in situ application in the oral environment.

### 3.6. Biocompatibility of ATDS Hydrogels

Acrylamide (AM), similar to THMA, is commonly used in the fabrication of adhesive hydrogels. This comparison allowed us to evaluate the relative cytotoxicity of THMA against a well-established monomer benchmark, demonstrating that the cytocompatibility of our THMA-based formulation is comparable to that of a widely used adhesive hydrogel material. To evaluate the biocompatibility of different monomer systems, the cytocompatibility of an AlgMA/AM/DMA/SCS-MA hydrogel containing 25% AM monomer (AADS hydrogel) was compared with that of the THMA-based ATDS hydrogel containing 25% THMA monomer, while maintaining the same contents of AlgMA, DMA, and SCS-MA. HaCaT cells were cultured with 24 h and 72 h hydrogel extracts, and complete medium served as the control.

**Figure 6:**
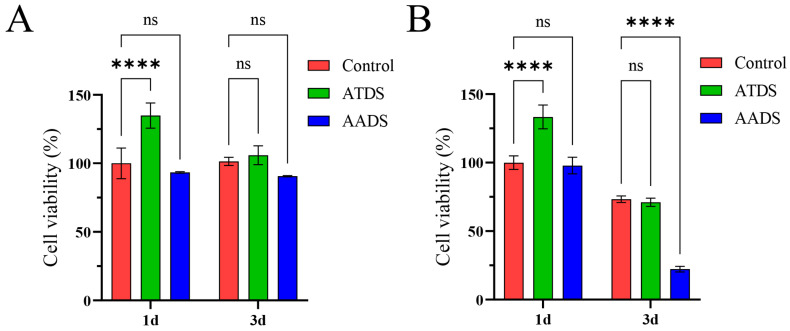
Cytotoxicity of hydrogels: (**A**) HaCaT cells cultured with 1-day extracts of hydrogels. (**B**) HaCaT cells cultured with 3-day extracts of hydrogels. **** *p* < 0.0001, ns: *p* > 0.05.

For the 24 h extracts (Figure 6A), both hydrogels exhibited good cytocompatibility. After 1 day of culture, cell viability in all groups exceeded 90% of that in the control. After 3 days, neither extract showed obvious cytotoxicity, with cell viabilities of 104% and 88%, respectively.

For the 72 h extracts (Figure 6B), both groups maintained good cell viability after 1 day of culture. However, after 3 days, the AADS hydrogel extract showed markedly reduced cell viability, indicating significant cytotoxicity.

### 3.7. Wound Healing of Diabetic Rat Oral Mucosa

**Figure 7:**
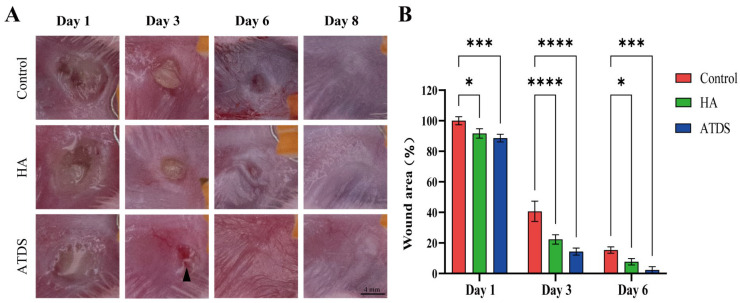
Wound closure rate: (**A**) Wound photograph. (**B**) Time-dependent change in wound closure rate. * *p* < 0.05, *** *p* < 0.001, **** *p* < 0.0001.

To evaluate wound healing in each group, wound images were captured at different time points using a digital camera (Nikon), and the relative wound area was quantified using ImageJ software (version: 1.51j8). As shown in Figure 7, the ATDS hydrogel significantly accelerated oral mucosal wound healing compared with the control and HA groups.

By day 3, approximately 40% of the wound area remained unhealed in the control group, whereas the residual wound area decreased to approximately 20% in the HA group and 15% in the ATDS group. On day 6, the ATDS-treated wounds were almost completely healed, with only ~3% residual wound area remaining, while the HA and control groups still exhibited approximately 8% and 15% unhealed areas, respectively. By day 8, re-epithelialization was nearly complete in all groups, with no visible exposed wounds. Notably, the ATDS group exhibited a smoother wound surface morphology more closely resembling normal oral mucosa.

Gross observations further showed that the ATDS hydrogel remained attached to the wound surface on day 3, whereas the HA hydrogel had completely disappeared, indicating superior retention stability of the ATDS hydrogel in the oral environment.

### 3.8. H&E Staining Results

**Figure 8:**
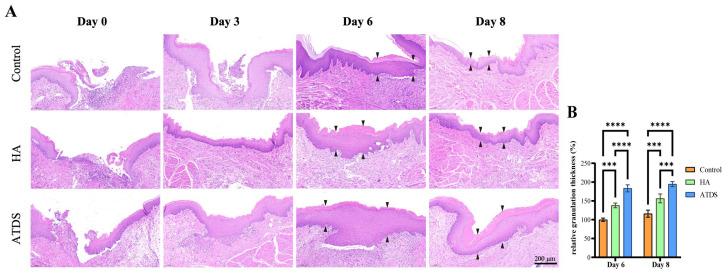
HE section staining: (**A**) H&E-stained images of wound tissues. (**B**) Relative epidermis thickness of wounds (normalized to the control group on day 6 as 100%). *** *p* < 0.001, **** *p* < 0.0001.

Hematoxylin and eosin (H&E) staining was performed to evaluate histological changes during wound healing, and granulation tissue thickness on days 6 and 8 was quantitatively analyzed using CaseViewer software (Figure 8).

As shown in Figure 8A, all groups exhibited exposed wounds without continuous epithelial coverage on day 1. Epithelial cells at the wound margins were sparsely distributed and had not yet migrated toward the wound center. The wound bed mainly consisted of loose fibrin and inflammatory exudates, with minimal collagen deposition or tissue remodeling, indicating an early inflammatory stage of healing.

By day 3, both the HA and ATDS groups had completed re-epithelialization, with newly formed tissue fully covering the wound surface, whereas the control group still lacked intact epithelial coverage. The HA hydrogel promoted wound repair to some extent because of its extracellular matrix-mimicking properties. Notably, the ATDS hydrogel exhibited thicker granulation tissue, likely owing to its strong tissue adhesion, protective barrier function, and the anti-inflammatory and pro-regenerative effects of SCS-MA.

On days 6 and 8, complete epithelial coverage was observed in all groups, indicating progression into the remodeling phase. Quantitative analysis of granulation tissue thickness demonstrated that both the HA and ATDS hydrogels significantly promoted tissue regeneration compared with the diabetic control group (Figure 8B). In particular, the thickness of the epidermis in the HA and ATDS groups increased by approximately 20% and 50%, respectively, relative to the control group.

Collectively, these results demonstrate that the ATDS hydrogel effectively promotes oral mucosal wound repair in diabetic rats.

### 3.9. CD31 and α-SMA Immunofluorescence Staining

**Figure 9:**
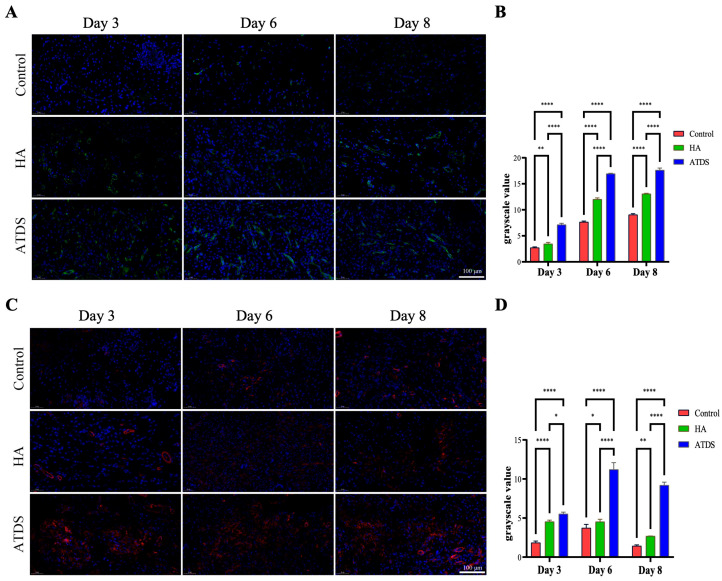
Immunofluorescence staining: (**A**) CD31 fluorescence (green) images. (**B**) Quantitative analysis of CD31. (**C**) α-SMA fluorescence (red) images. (**D**) Quantitative analysis of α-SMA. * *p* < 0.05, ** *p* < 0.01, **** *p* < 0.0001.

CD31 and α-SMA are widely used markers for evaluating angiogenesis and vascular maturation. CD31 is mainly expressed in newly formed blood vessels, whereas α-SMA is associated with mature vasculature. Immunofluorescence staining results (Figure 9A,B) showed that the ATDS group exhibited abundant CD31 expression on day 3, indicating enhanced neovascularization at the wound site. The HA group also showed moderate CD31 expression, whereas the DM group exhibited minimal expression, likely because persistent inflammation impaired angiogenesis under diabetic conditions.

By day 8, CD31 expression decreased in all groups, while α-SMA expression progressively increased from day 3 to day 8, reflecting vascular maturation during wound remodeling. Notably, α-SMA expression in the ATDS group reached a relatively high level by day 6 and consistently exceeded that of the other groups (Figure 9C,D). These findings demonstrate that the ATDS hydrogel effectively promotes angiogenesis and vascular maturation during diabetic oral mucosal wound healing.

### 3.10. ATDS Hydrogel Reduces Inflammatory Response in Diabetic Rat Oral Mucosa Wounds

**Figure 10:**
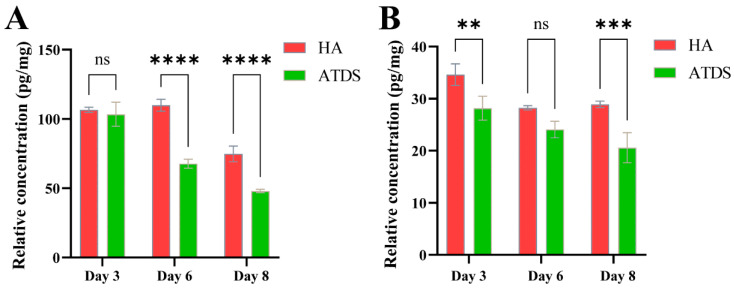
Relative levels of inflammatory factors: (**A**) TNF-α levels; (**B**) IL-1β levels. ** *p* < 0.01, *** *p* < 0.001, **** *p* < 0.0001, ns: *p* > 0.05.

To evaluate the anti-inflammatory effects of the ATDS hydrogel, the levels of the pro-inflammatory cytokines TNF-α and IL-1β in wound tissue homogenates were quantified by ELISA. As shown in Figure 10A, TNF-α expression gradually decreased during the healing process from day 3 to day 8. Compared with the HA group, the ATDS hydrogel significantly reduced TNF-α levels on days 6 and 8. Similarly, IL-1β expression was also markedly downregulated in the ATDS-treated group (Figure 10B).

These results indicate that the ATDS hydrogel effectively suppresses inflammatory responses in diabetic oral mucosal wounds, thereby contributing to an improved wound-healing microenvironment.

## 4. Discussion

The delayed healing of oral mucosal wounds in diabetic patients involves complex mechanisms, primarily encompassing multiple pathological factors such as vascular dysfunction, persistent inflammation, and restricted epithelial regeneration under a hyperglycemic microenvironment [13]. To address these challenges, we developed a multifunctional photocurable hydrogel (ATDS) composed of methacryloylated sulfonated chitosan (SCS-MA), methacrylated alginate (AlgMA), N-acryloyl tris(hydroxymethyl)aminomethane (THMA), and 3-methacryloyl dopamine (DMA). Through the synergistic integration of these components, the hydrogel exhibited favorable biocompatibility, wet adhesion, mechanical strength, and bioactivity, making it suitable for diabetic oral mucosal repair.

SCS-MA retained the biological activity of sulfonated chitosan while introducing methacryloyl groups for rapid photocrosslinking, thereby avoiding the limitations of conventional chemical crosslinkers, such as cytotoxicity and slow gelation [47,48]. Upon UV irradiation, the ATDS hydrogel rapidly formed a stable porous network structure. SEM analysis revealed an interconnected porous architecture with an average pore size of approximately 50 μm, which is favorable for nutrient transport, cell migration, and tissue ingrowth while maintaining a moist wound microenvironment [49]. Rapid in situ gelation is particularly advantageous in the oral cavity, where saliva flow and mechanical disturbance can readily displace conventional materials.

Wet adhesion and mechanical stability are essential for oral wound dressings. THMA contributed abundant hydroxyl groups that enhanced tissue adhesion; however, THMA-based hydrogels alone generally exhibit insufficient mechanical strength [16,20]. Incorporation of AlgMA not only improved the biocompatibility but also significantly enhanced the tensile strength and extensibility of the hydrogel through a double-network toughening mechanism [22,50,51]. AlgMA acts as a macromolecular crosslinker via photo-initiated polymerization, covalently incorporating its flexible chains into the rigid network. This rigid–flexible synergy provides strength, while hydrogen bonds on AlgMA chains serve as sacrificial bonds to dissipate energy and delay network failure. Compared with the TDS hydrogel lacking AlgMA, the ATDS hydrogel exhibited a markedly higher tensile strength (50 kPa vs. 10.31 kPa) and an approximately 10-fold increase in elongation at break, indicating improved resistance to mechanical deformation in the oral environment. In addition, increasing the THMA content from 15% to 25% enhanced the shear adhesive strength from 0.494 MPa to 0.605 MPa, representing a 22% increase. Compared with medical fibrin glue (<4 kPa) [52] and previously reported acrylic acid/catechol adhesive hydrogels [53], the ATDS hydrogel demonstrated substantially stronger adhesion to pig skin.

Retention stability is another critical factor for oral wound repair because conventional oral formulations are rapidly removed by saliva and often remain effective for less than 1 h [54]. In contrast, the ATDS hydrogel reached swelling equilibrium within 24 h and maintained structural stability thereafter, enabling prolonged coverage of the wound surface. SEM analysis revealed intimate contact between the hydrogel and palatal mucosal tissue with minimal interfacial gaps. Furthermore, in vivo observations showed that the ATDS hydrogel remained attached to oral wounds for more than 72 h, whereas the HA hydrogel had completely disappeared by day 3. These findings indicate that the ATDS hydrogel possesses favorable tissue conformability and retention capability in the moist oral environment, thereby providing sustained support for tissue repair.

Because precursor solutions of in situ curing hydrogels directly contact wound tissues before gelation, monomer biocompatibility is particularly important. The higher cytotoxicity associated with AM suggests that it is less suitable for in situ oral wound applications, whereas the THMA-based ATDS hydrogel demonstrated superior biosafety.

From a biological perspective, SCS-MA not only contributed to the structural properties of the hydrogel but also endowed the material with heparin-like bioactivity, including anti-inflammatory and pro-angiogenic effects. In the diabetic oral mucosal defect model, ATDS hydrogel treatment significantly accelerated wound closure and promoted granulation tissue formation, both of which are key indicators of wound healing [55,56]. Compared with the control and HA groups, the ATDS-treated group exhibited smaller residual wound areas on days 3 and 6, as well as more extensive granulation tissue formation on days 6 and 8, confirming its effectiveness in alleviating diabetes-impaired wound healing. Furthermore, immunofluorescence analysis demonstrated increased expression of CD31 and α-SMA in the ATDS group, indicating enhanced angiogenesis. Since impaired neovascularization is a hallmark of diabetic wounds [57], the improved vascular regeneration likely contributed to the accelerated tissue repair observed in this study.

Persistent inflammation is another major barrier to diabetic wound healing [58,59]. Excessive production of pro-inflammatory cytokines such as TNF-α and IL-1β prolongs inflammatory responses and disrupts tissue regeneration. In this study, ATDS hydrogel treatment significantly reduced TNF-α and IL-1β levels in wound tissues, indicating effective modulation of the inflammatory microenvironment. Combined with its pro-angiogenic activity, these results suggest that SCS-MA-containing hydrogels may promote diabetic wound healing through coordinated regulation of inflammation and angiogenesis.

Overall, the ATDS hydrogel demonstrated favorable structural characteristics, wet adhesion, mechanical stability, and therapeutic efficacy in diabetic oral mucosal repair, highlighting its potential as a multifunctional wound dressing for oral applications. Nevertheless, several limitations remain. The long-term adhesion behavior of the hydrogel under continuous salivary erosion requires further optimization, and the molecular mechanisms underlying its immunomodulatory and pro-angiogenic effects warrant deeper investigation. In addition, future studies should evaluate large-scale manufacturing feasibility, long-term storage stability, and clinical translational potential.

## Figures and Tables

**Table 1:** Preparation of the hydrogel.

	0.75 wt%AlgMA (mL)	THMA (g)	DMA (mg)	SCS-MA (mg)	LAP (mg)
TDS	-	0.5	10	15	1
15 wt%ATDS	2	0.3	10	15	1
25 wt%ATDS	2	0.5	10	15	1

## Data Availability

Data are contained within this article.

## Abbreviations

The following abbreviations are used in this manuscript:

HA	Hyaluronic acid
THMA	N-acryloyl tris(hydroxymethyl)aminomethane
SCS	Sulfonated chitosan
AlgMA	Methacrylated alginate
DMA	3-methacryloyl dopamine
SCS-MA	Methacryloylated sulfonated chitosan
AM	Acrylamide
STZ	Streptozotocin
ELISA	Enzyme-linked immunosorbent assay
SEM	Scanning electron microscopy
PBS	Phosphate-buffered saline
H&E	Hematoxylin and eosin
